# Involvement of brain-derived neurotrophic factor signaling in the pathogenesis of stress-related brain diseases

**DOI:** 10.3389/fnmol.2023.1247422

**Published:** 2023-09-14

**Authors:** Tadahiro Numakawa, Ryutaro Kajihara

**Affiliations:** ^1^Department of Cell Modulation, Institute of Molecular Embryology and Genetics, Kumamoto University, Kumamoto, Japan; ^2^Department of Biomedical Laboratory Sciences, Faculty of Life Science, Kumamoto University, Kumamoto, Japan

**Keywords:** BDNF, TrkB, major depressive disorder, Alzheimer’s disease, glucocorticoid

## Abstract

Neurotrophins including brain-derived neurotrophic factor, BDNF, have critical roles in neuronal differentiation, cell survival, and synaptic function in the peripheral and central nervous system. It is well known that a variety of intracellular signaling stimulated by TrkB, a high-affinity receptor for BDNF, is involved in the physiological and pathological neuronal aspects via affecting cell viability, synaptic function, neurogenesis, and cognitive function. As expected, an alteration of the BDNF/TrkB system is suspected to be one of the molecular mechanisms underlying cognitive decline in cognitive diseases and mental disorders. Recent evidence has also highlighted a possible link between the alteration of TrkB signaling and chronic stress. Furthermore, it has been demonstrated that downregulation of the BDNF/TrkB system and chronic stress have a role in the pathogenesis of Alzheimer’s disease (AD) and mental disorders. In this review, we introduce current evidence showing a close relationship between the BDNF/TrkB system and the development of cognition impairment in stress-related disorders, and the possible contribution of the upregulation of the BDNF/TrkB system in a therapeutic approach against these brain diseases.

## 1. Introduction

Changed levels of neurotrophins have long been implicated in the pathophysiology of brain diseases including psychiatric diseases and neurodegenerative diseases. The neurotrophins, including nerve growth factor (NGF), brain-derived neurotrophic factor (BDNF), neurotrophin-3 (NT-3), and neurotrophin-4/5, were first identified as survival promoting factors, in both the peripheral nervous system and central nervous system (CNS; Skaper, 2018). It has been demonstrated that neurotrophins, especially BDNF, are key molecules which are essential for neuroprotection, cell differentiation, and regulation of synaptic plasticity (Numakawa and Kajihara, 2023). These neuronal aspects are exerted via activation of three members of the tropomyosin-related kinase family of receptor tyrosine kinases, including TrkA (specific for NGF), TrkB (for BDNF and NT-4), and TrkC (for NT-3) although the p75 neurotrophin receptor (p75NTR) was first identified as a low affinity receptor for all the neurotrophins.

Recently, there is a growing body of evidence that the altered levels of neurotrophins and their receptors, particularly BDNF/TrkB system, are correlated with major depressive disorder (MDD) and AD (Autry, 2022; Kumari et al., 2023). Because individuals with depressive disorders display cognitive impairments (Airaksinen et al., 2004), it is suggested that downregulation of BDNF/TrkB system is the major mechanism for the pathogenesis of MDD, while stress including increased levels of glucocorticoids caused by overactivation of hypothalamic–pituitary–adrenal (HPA) axis, affects expression/function of the BDNF/TrkB system (Numakawa et al., 2014).

Interestingly, there is a possible contribution of glucocorticoid stress to the pathogenesis of AD (Caruso et al., 2019). Evidence suggest that glucocorticoids affect the accumulation of amyloid β (Aβ) and hyperphosphorylation of tau, that are key pathological features of AD (Zhuravleva et al., 2021). As expected, epidemiological and clinical studies have demonstrated possible association among glucocorticoid stress, cognitive decline, and AD development (Pomara et al., 2003; Gil-Bea et al., 2010).

Here, we show current evidence showing a close relationship between the downregulation of BDNF/TrkB system and the depression models after the glucocorticoid stress. Furthermore, contribution of the altered BDNF/TrkB system in the development of cognition decline in the pathogenesis of AD, as one of the stress-related disorders.

## 2. BDNF/TrkB-mediated intracellular signaling

BDNF, which is the most intensively studied neurotrophin, exerts positive effects including enhancement of synaptic function and neuroprotection against neuronal damages. In general, it has been considered that the positive influence of BDNF in neurons is via activating TrkB, a high affinity receptor for mature BDNF. The dimerization and autophosphorylation of intracellular tyrosine residues of TrkB is required for activation of several intracellular signaling (Kaplan and Miller, 2000). The mitogen-activated protein kinase (MAPK), phospholipase Cγ (PLCγ), phosphatidylinositol3-kinase (PI3K), guanosine triphosphate hydrolases (GTP-ases) of the Ras homolog (Rho) gene family pathways are stimulated after the activation (phosphorylation) of TrkB (Huang and Reichardt, 2003; Minichiello, 2009; Gonzalez et al., 2016).

**Figure 1:**
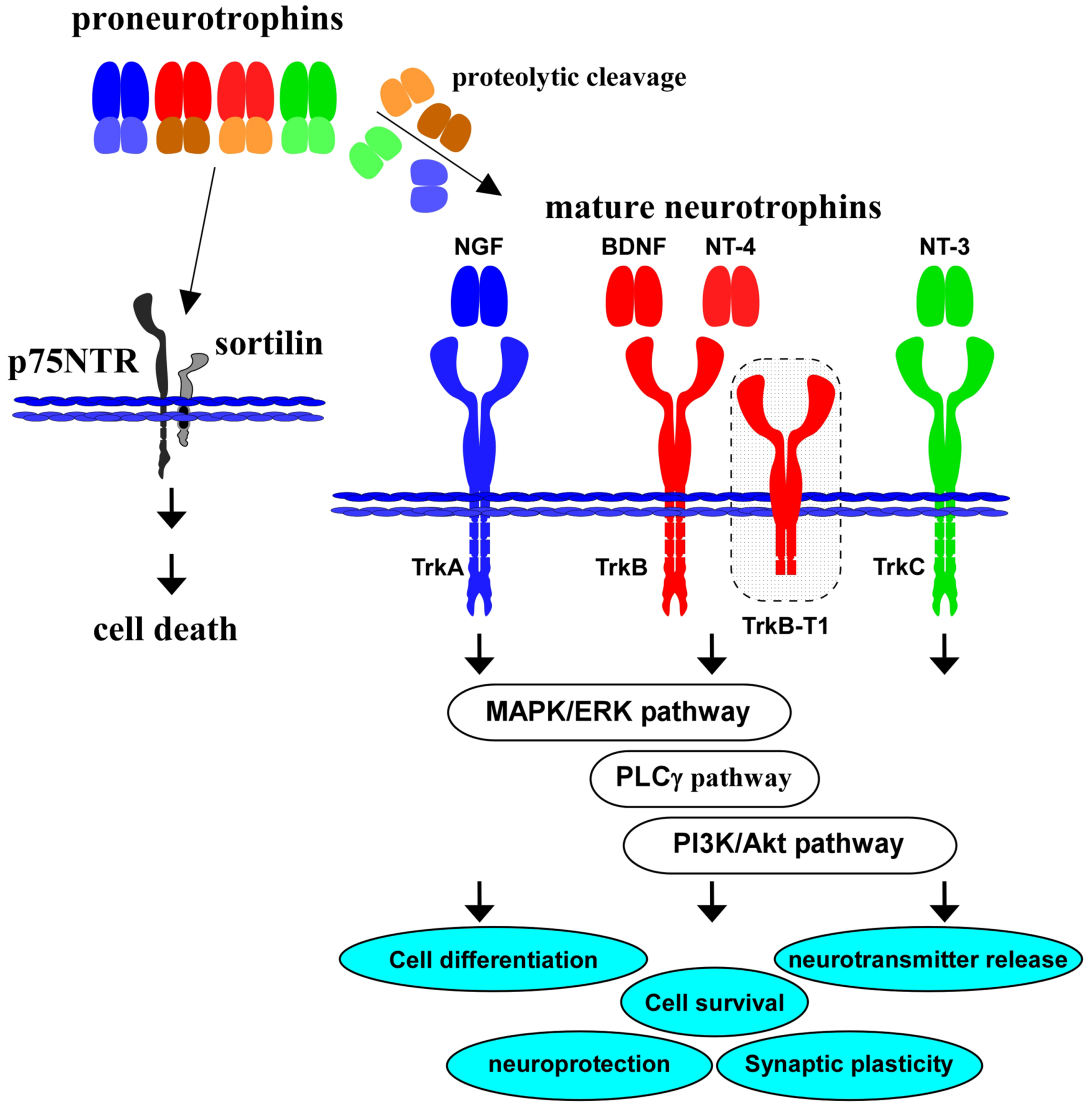
Proneurotrophins, mature neurotrophins and receptors. All neurotrophins, including brain-derived neurotrophic factor (BDNF), nerve growth factor (NGF), neurotrophin-3 (NT-3), and neurotrophin-4 (NT-4) are produced firstly as precursor proneurotrophins. Proneurotrophins bind to p75NTR (with a high affinity) and sortilin (co-receptor), resulting in neuronal cell death. Activation of TrkB, a high affinity receptor for mature BDNF after proteolytic cleavage of proBDNF, and downstream intracellular signaling (MAPK/ERK-, phospholipase Cγ (PLCγ)-, PI3K/Akt-pathways) are involved in cell differentiation, cell survival, synaptic plasticity, and neuroprotection in the CNS. Truncated isoform TrkB.T1 also contributes to action of BDNF. NGF binds to TrkA with a high affinity, similarly, NT-4 binds to TrkB, and NT-3 to TrkC, respectively.

The MAPK pathway is involved in the activation of cAMP response element-binding protein (CREB) and extracellular-signal-regulated kinase 1/2 (ERK 1/2; Finkbeiner et al., 1997; Xing et al., 1998). Previously, we reported that the MAPK/ERK signaling contributed to the upregulation of synaptic protein expression by BDNF in cultured cortical neurons (Kumamaru et al., 2011). It is well known that BDNF increases intracellular Ca^2+^ concentration in neuronal cells via the PLCγ-dependent pathway. Protein kinase C (PKC), Ca^2+^-calmodulin-dependent protein kinases (CaMKs), and production of 1,2-diacylglycerol (DAG) are regulated by the PLCγ pathway (Alcántara et al., 1997; Minichiello, 2009). In cultured cortical neurons, glutamate, an excitatory neurotransmitter, is released via activation of the PLCγ pathway after exposure to BDNF (Numakawa et al., 2009). It has been also demonstrated that PI3K/Akt pathway contribute to the pro-survival, anti-apoptotic, and synaptic plasticity (Park and Poo, 2013; Baydyuk and Xu, 2014; Gonzalez et al., 2016; Rai et al., 2019). In these neuronal responses, BDNF/TrkB system including downstream intracellular signaling has pivotal roles (Numakawa et al., 2010; Figure 1).

## 3. TrkB truncated isoforms, and p75NTR, a low affinity receptor

TrkB truncated isoforms also contribute to the biological effects of BDNF. Isoforms including TrkB.T1, one of TrkB isoforms whose catalytic kinase domain is lacking, is involved in changed BDNF action through ligand scavenging effect. As expected, evidence suggests possible involvement of TrkB truncated isoforms in the pathogenesis of neurological disorders including AD and mood disorders (see Tessarollo and Yanpallewar, 2022). In addition to TrkB.T1, splice variants of the full-length TrkB, TrkB. Shc, and TrkC.T1 in the CNS have been reported (see Gupta et al., 2013). In ferret visual cortical slices, increased proximal dendritic branching was induced by transfection of full-length TrkB, in contrast, net elongation of distal dendrites was caused by truncated TrkB transfection (Yacoubian and Lo, 2000). Michaelsen et al. (2010) reported deficits in the long-term potentiation and long-term depression in the hippocampus of the TrkB.T1 overexpression transgenic mice (Michaelsen et al., 2010).

Although p75NTR, a low affinity common receptor for neurotrophins, does not have catalytic activity (Liepinsh et al., 1997), several interactors for the p75NTR-mediated intracellular signaling have been reported (Mukai et al., 2000; Hempstead, 2002; Becker et al., 2018). For example, Yamashita et al. (1999) found the increased neurite elongation through interaction between p75NTR and Ras homolog gene family member A (RhoA; Yamashita et al., 1999). Ye et al. (1999) reported regulation of the nuclear factor-kB signaling via interaction of p75NTR with tumor necrosis factor receptor-associated factors (TRAF) family proteins (Ye et al., 1999).

Importantly, neurotrophins, including NGF and BDNF, are firstly translated as precursors (proneurotrophins, Figure 1), and are subsequently cleaved to mature molecules (mature NGF, mature BDNF, see Figure 1). These small mature proteins have a high affinity for each specific Trk receptor. On the other hand, there is a growing body of evidence that proneurotrophins (including proBDNF) bind to p75NTR with high affinity and that the neuronal aspects including cell survival and synaptic plasticity are regulated negatively by the proneurotrophins/p75NTR-mediated signaling (Teng et al., 2005; Yang et al., 2014; Meeker and Williams, 2015). For example, Nykjaer et al. (2004) reported action of sortilin as a co-receptor for proNGF in the proNGF/p75NTR-mediated neuronal cell death (Nykjaer et al., 2004). Sortilin also recognize the pro-domain structure of proBDNF and has a role in the cell death induction (He and Garcia, 2004; Nykjaer et al., 2004). It is possible that expression balance in the full-length/truncated forms of TrkB, TrkB/p75NTR, or expression pattern of interactors for p75NTR may affect the neuronal cell fate, and contribute to the pathogenesis of brain diseases including depression and AD.

## 4. BDNF-related signaling and depression

**Figure 2:**
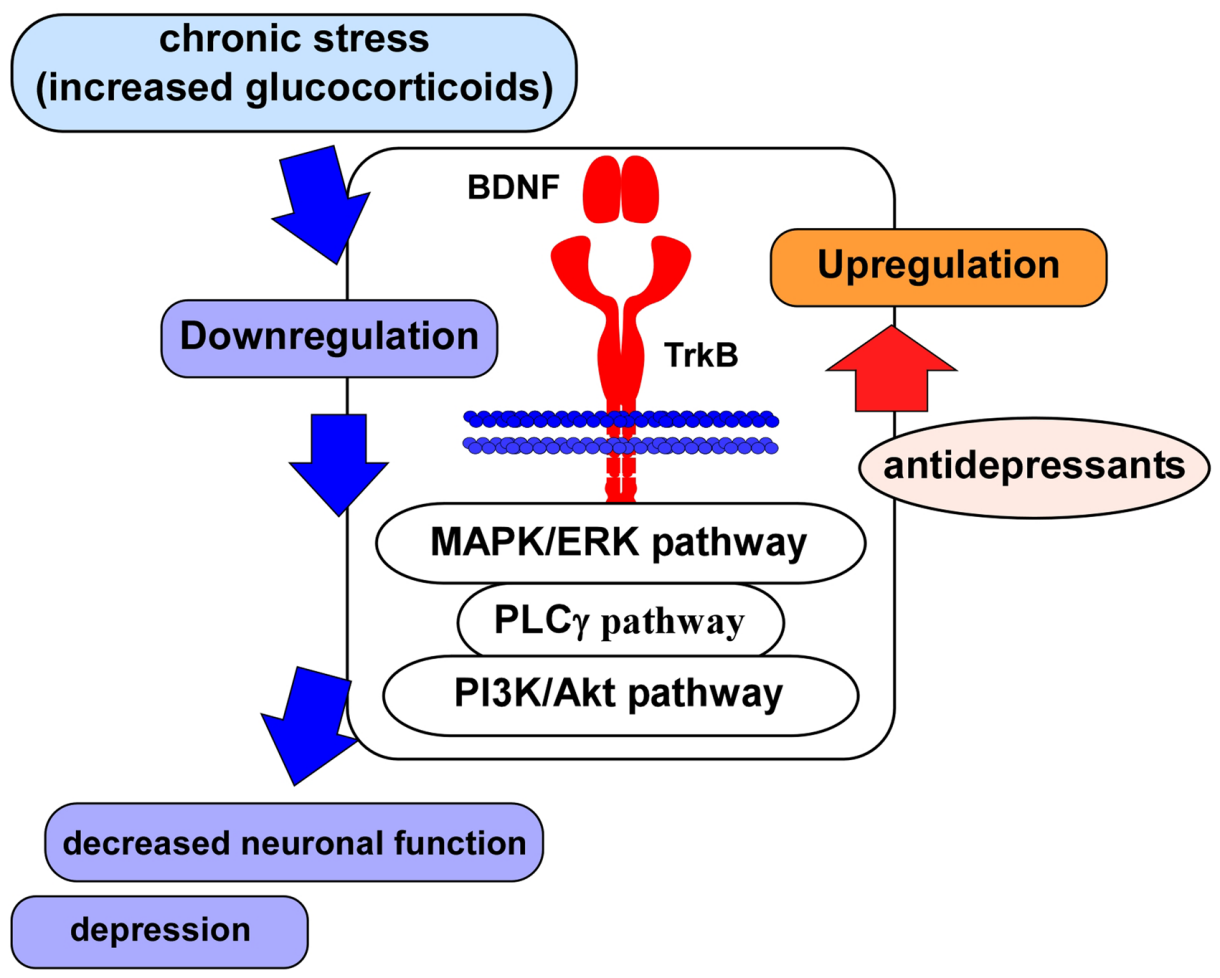
Glucocorticoid stress and BDNF/TrkB system. Impairment of neuronal function associated with depression may be caused via the downregulation of BDNF/TrkB system and its downstream signaling (MAPK/ERK-, PLCγ-, PI3K/Akt-pathways) after the exposure to glucocorticoid stress. Antidepressants induce upregulation of the BDNF/TrkB system, leading to improvement of neuronal function.

As mentioned above, the BDNF/TrkB system contributes to maintenance of neuronal cells, synaptic function and neuroprotection. Therefore, downregulation of the BDNF/TrkB system is critical for pathophysiology of brain diseases such as MDD and AD. As expected, studies suggest a close relationship among changed activity and/or content of BDNF, depressive behavior, and antidepressant action (Figure 2). Guilloux et al. (2012) reported BDNF-related dysfunctions in the amygdala of female subjects with depression (Guilloux et al., 2012). Recent postmortem analysis also revealed decreased plasma BDNF levels in depressed patients compared with those in controls (Gadad et al., 2021). Therefore, BDNF-related system is an important strategy for the development of novel antidepressant medications (Numakawa et al., 2014). Furthermore, it is a serious problem that the unpredictability of antidepressant responses is uncertain in depressed patients. Using genetic polymorphisms, Santos et al. (2023) have reported a relationship between antidepressant treatment phenotypes and genetic variants in neuroplasticity-related genes including BDNF and TrkB (Santos et al., 2023). They carried out genotyping 26 polymorphisms in genes of BDNF, NTRK2 (for TrkB), NGFR (p75NTR), CREB1, glycogen synthase kinase-3β (GSK3B), protein kinase B (Akt), MAPK1, mTOR, phosphatase and tensin homolog (PTEN), activity-regulated cytoskeletal-associated protein (ARC), and synapsin I (SYN1) in 80 patients with MDD. It was revealed that the treatment-resistant depression was associated with BDNF rs6265, SYN1 rs1142636, and PTEN rs12569998 SNP. Furthermore, the disease relapse was associated with MAPK1 rs6928 and GSK3B rs6438552 polymorphisms (Santos et al., 2023). Recently, with drug-naïve patients with first-episode schizophrenia, an examination on correlations of BDNF, CREB, PI3K, and Akt protein levels in the peripheral blood with depressive emotion/impulsive behaviors of schizophrenia has been investigated (Li et al., 2023). Interestingly, the study results showed that levels of BDNF, CREB, and PI3K (not Akt) were correlated negatively with depressive emotion and impulsive behaviors (Li et al., 2023).

In addition to the contribution of neurons, that of microglial function in the effectiveness of treatments against depression was also demonstrated. Using mice that received chronic mild stress (CMS), Zhang et al. (2023) investigated the effects of Akebia saponin D (ASD) administration on microglial phenotype, neurogenesis in the hippocampus, and behaviors (Zhang et al., 2023). Depressive-like behaviors (analyzed by sucrose preference test (SPT), forced swimming test (FST), and open field test (OFT)) observed after CMS were reversed by 3-week treatment with ASD. They also suggest that that ASD acts via activating the peroxisome proliferator-activated receptor-gamma (PPAR-γ) to induce pro-neurogenic microglia that induce upregulation of BDNF, and neurogenesis in the hippocampal dentate gyrus (DG; Zhang et al., 2023). Endogenous lipopolysaccharide (LPS) is produced in the gut which is one of the important factors affecting the development of neuroinflammation and/or mental disorders including depression (Ait-Belgnaoui et al., 2012; Suda and Matsuda, 2022). Lu et al. (2022) have reported a relationship between BDNF and an antidepressant effect by LPS. Lower levels of microglia in the DG in depressed mice were reversed by a single low dose of LPS. Markedly, blockade of BDNF signaling (by knock-in of the mutant BDNF Val68Met allele, a neutralizing antibody for BDNF, and an inhibitor for TrkB) blocked the antidepressant effect achieved by LPS application in chronically stressed mice (Lu et al., 2022). Astroglia is also major population in the CNS and is involved BDNF-related molecules. Tokunaga et al. (2022) examined the relationship among connexin 43 (Cx43) expression and BDNF induction and antidepressants in astrocytes (Tokunaga et al., 2022). They showed that amitriptyline, one of tricyclic antidepressants, enhanced expression of BDNF in Cx43-knockdown astrocytes, and lysophosphatidic acid receptor (1/3) and activation of ERK signaling is involved in the enhanced BDNF expression by amitriptyline (Tokunaga et al., 2022). Using the depression mouse models caused by chronic adreno-cortico-tropic-hormone (ACTH)- and chronic unpredictable mild stress (CUMS), fast-acting (1 h) antidepressant effects of ketamine has been reported (Ma et al., 2022). The ketamine injection induced upregulation of glutamate transporter-1(GLT-1), glial fibrillary acidic protein (GFAP, a marker for astroglia), and BDNF, and reduced eukaryotic elongation factor 2 phosphorylation in the prelimbic prefrontal cortex in addition to reduced glutamate responses to synaptic stimulation in the cortical excitatory neurons. Interestingly, these changes by acute ketamine injection still occurred after GLT-1 knockdown, suggesting no involvement of GLT-1 upregulation and distinct contributions from astrocytes and neurons in the action of ketamine (Ma et al., 2022). As expected, recent evidence demonstrates an important of glial dysfunction in the pathogenesis of emotional diseases including depression and of therapeutic strategies involving astrocytes via BDNF-dependent mechanisms (Koizumi, 2021).

## 5. Role of BDNF-related intracellular signaling in action of antidepressants

It has been demonstrated that different neurotransmitter systems are involved in the pathogenesis of depression and the deficits in the neurotransmitter systems are restored by a variety of antidepressant treatment (Taylor et al., 2005). The influence of antidepressants on the BDNF/TrkB system was first demonstrated by Nibuya et al. (1995). They found that chronic administration of various antidepressant drugs induced a significant increase in mRNA levels of BDNF in the hippocampus, thereby providing neuroprotection against the detrimental effects of stress on neurons (Nibuya et al., 1995). Importantly, as evidence suggest depression coexists with epilepsy, possible interactions between antiepileptic drugs and antidepressant has been shown (Borowicz-Reutt, 2021). For example, the mechanism of action of ketamine, which is used medically as anesthetic, is intensively investigated because of its antidepressant potential and association with BDNF/TrkB system (Rantamäki, 2019). Using CUMS mouse model, a recent study has demonstrated that depressive-like behaviors in the animals were improved by repeated s-ketamine application (Liu et al., 2023). In SPT, CUMS mice exhibited a significantly reduced sucrose preference compared with those in the control animals. Interestingly, s-ketamine administration restored the reduced sucrose preference caused by CUMS. In addition, CUMS-induced hippocampal elevation of pCaMKIIα and downregulation of BDNF, pTrkB, and mTOR were all reversed by s-ketamine administration (Liu et al., 2023).

Remarkably, it has been suggested that combining low-frequency electromagnetic fields (ELF-EMF) and ketamine, which are recognized as effective treatment against depression-like behavior, respectively, has adverse effects (Salari et al., 2023). Salari et al. (2023) performed behavioral, histological and molecular study using male rats received ketamine treatment and/or ELF-EMF following 3 weeks chronic unpredictable stress. It was revealed that combining treatment of ELF-EMF and ketamine increased depression-like behavior, degenerated hippocampal and cortical pyramidal neurons. Furthermore, decreased BDNF and increased caspase-3 levels were also confirmed (Salari et al., 2023). The beneficial effect of (2R,6R)-hydroxynorketamine [(2R,6R)-HNK], a metabolite of ketamine, has been also demonstrated. Using rat model of post-traumatic stress disorder (PTSD) caused by single prolonged stress and electric foot shock, effects of (2R,6R)-HNK administration (in the nucleus accumbens by microinjection) were examined (Gou et al., 2023). (2R,6R)-HNK administration improved exploration and depression-linked behaviors in the PTSD rats. Furthermore, (2R,6R)-HNK treatment also reversed downregulation of BDNF, p-mTOR, and PSD95 observed in the PTSD rats (Gou et al., 2023). Using female Institute of Cancer Research mice, it has been shown a synergistic antidepressant effect of iron co-applied with citalopram or imipramine (Kukuia et al., 2023). Kukuia et al. (2023) examined depression-like symptoms (by FST, tail suspension test, and open space swim test) when mice received the combination treatment of a sub-therapeutic dose of iron with classical antidepressants (citalopram or imipramine). It was revealed that the combination of iron with antidepressants achieved more rapid and superior antidepressant-like effect than the iron, citalopram or imipramine alone. Furthermore, they confirmed increased serum BDNF levels and dendritic spines density in the hippocampus.

Traditionally, it has been widely believed that typical slow-acting antidepressants including selective serotonin reuptake inhibitors (SSRIs) exert their effects through monoamine transporters, while rapid-acting antidepressants such as ketamine act on glutamate NMDA receptors, although both pathways ultimately lead to the activation of BDNF/TrkB signaling (Casarotto et al., 2022). However, the recent studies suggest that their therapeutic benefits may not be solely mediated by these classical pathways. Casarotto et al. (2021) recently found that both typical and rapid-acting antidepressants directly bind to the TrkB receptors, leading to an allosteric potentiation of TrkB signaling (Casarotto et al., 2021). In the study, they also showed that mutation of the antidepressant-binding motif in TrkB impaired behavioral and plasticity-promoting reactions to antidepressants (Casarotto et al., 2021). In addition, it is suggested that antidepressant binding can stabilize and sensitize the TrkB receptors, facilitating the BDNF-mediated TrkB signaling (Castrén and Monteggia, 2021). These findings support that TrkB serves as the direct target for antidepressant drugs, mediating their therapeutic effects.

Importantly, the effect of phytochemicals that improve deficits in the system of neurotrophic factors on the pathogenesis of depression has been focused (see Wang et al., 2023). In addition to BDNF, changed levels of glial cell line-derived neurotrophic factor (GDNF), vascular endothelial growth factor (VEGF), and NGF in the depression brains were reported (Wang et al., 2023). Using animal models, antidepressant action by treatment with phytochemicals based on these neurotrophic factors have been demonstrated (see Wang et al., 2023).

## 6. Relationship between BDNF, glucocorticoid stress, and depression

**Figure 3:**
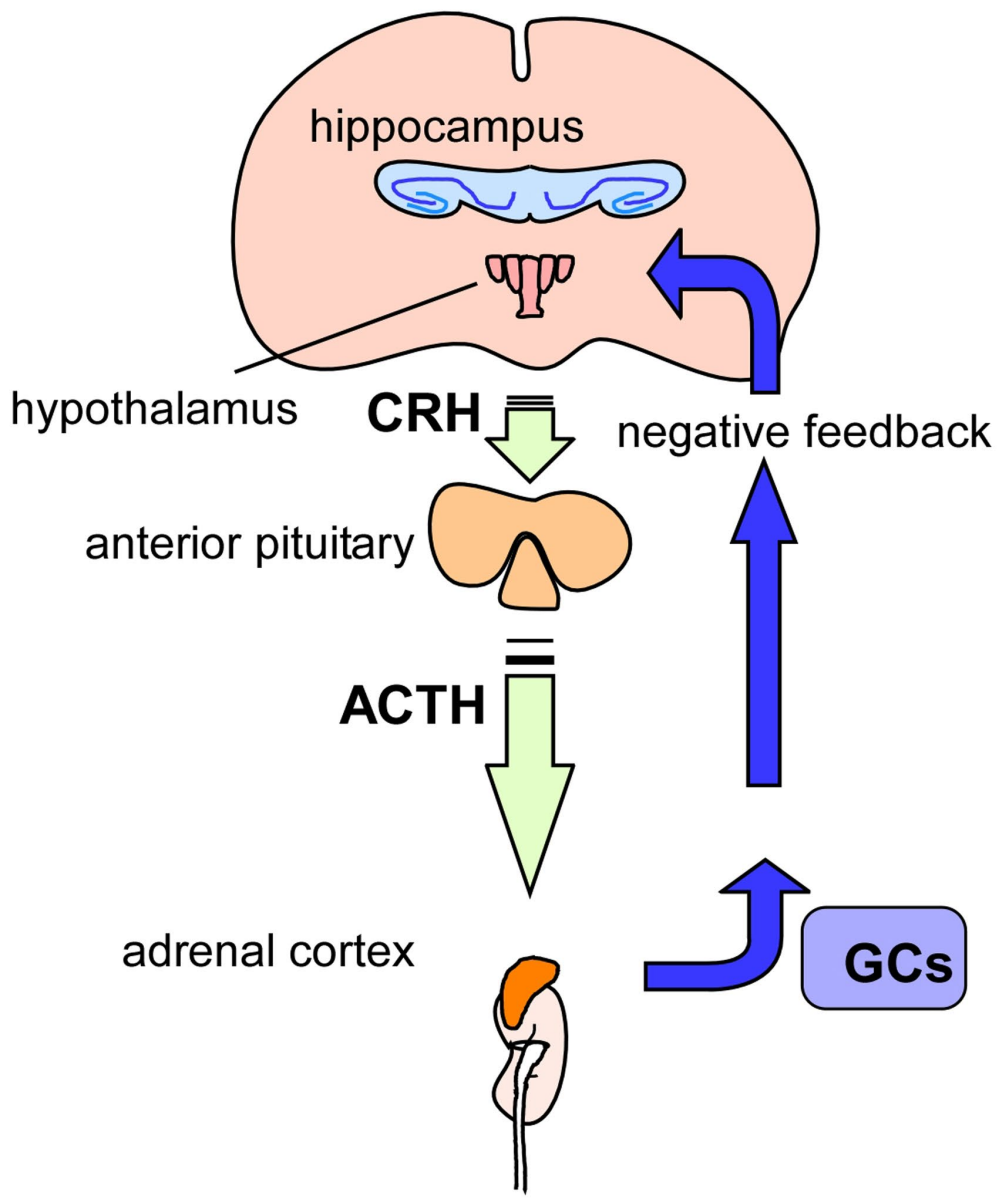
Glucocorticoids and HPA-axis. Hypothamic-Pituitry-Adrenal-axis (HPA-axis) regulates blood levels of glucocorticoids (GCs) to cope with stressful conditions. Released GCs from the adrenal glands, on top of the kidneys, also has a role in regulation of GCs levels via negative-feedback action in the HPA-axis. It has been suggested that chronic stress causes hyperactivation of HPA-axis, resulting in increased levels of GCs, contributing to the pathogenesis of depression. GCs, glucocorticoids; CRH, corticotropin-releasing hormone; ACTH, adrenocorticotropic hormone.

It has been well demonstrated that glucocorticoids have a role in the pathogenesis of depression (Table 1). Increased levels of glucocorticoids due to hyperactivation of Hypothamic-Pituitry-Adrenal-axis (HPA-axis, see Figure 3) are considered to be one of the risk factors for depression (Numakawa et al., 2014). Recently, the effect of chronic oxytocin (OT) administration using a female mouse depression model induced by dexamethasone (DEX, a synthetic glucocorticoid) has been investigated (Mori et al., 2022). After administration of vehicle, DEX, or OT + DEX daily for 8 weeks, the animals were assessed for anxiety-and depression-like behavior tests (OFT, elevated plus maze test (EPMT), and FST) and for expression of pCREB and BDNF in the hippocampus. As expected, mice exposed to DEX exhibited increased anxiety-and depression-like behaviors and plasma levels of corticosterone. Importantly, simultaneous OT application inhibited effects by DEX, and induced upregulation of hippocampal pCREB and BDNF levels (Mori et al., 2022). Recently, antidepressant properties of *Pedicularis resupinata* (one of Korean oriental medicines) was investigated using corticosterone (CORT)-induced depression mouse model (Lim et al., 2022). Although depressive-like behaviors, which were evaluated with the OFT, SPT, passive avoidance test, tail suspension test, and FST, were observed in male mice received injection of CORT, the CORT-induced depressive-like behaviors were improved by orally *P. resupinata* extract (PRE) administration. Importantly, reduced BDNF, and increased total glucocorticoid receptor (GR) and phosphorylated GR (at serine 211) in depression animals were also reversed by PRE (Lim et al., 2022), suggesting an antidepressant effect of *P. resupinata* through the BDNF-and/or GR-mediated molecular mechanism.

**Table 1:** BDNF/TrkB system and depression models.

Experimental depression models	Treatments	Behaviors, neurotrophic effects including BDNF action	References
Female mouse depression model induced by dexamethasone (DEX, a synthetic glucocorticoid)	Oxytocin administration daily for 8 weeks	Improvement of anxiety-and depression-like behaviors, and upregulation of hippocampal pCREB and BDNF levels	Mori et al. (2022)
Corticosterone (CORT)-induced depression mouse model	Oral *P. resupinata* extract (PRE, one of Korean oriental medicines) administration	Reduced depressive-like behaviors, upregulation of BDNF, and downregulation of both total GR and pGR (at serine 211)	Lim et al. (2022)
Male C57BL/6 mice received a chronic stress paradigm comprising of intermittent social defeat and overcrowding	Administration of SAFit2 (a selective inhibitor of FKBP51, 20 mg/kg; i.p.) twice a day	Prevention of stress-induced social avoidance, and increased anxiety	Codagnone et al. (2022)
Depression mice established by chronic corticosterone (CORT) injection	A single administration (1 h) of formononetin (FMN, 20 mg/kg) by oral gavage	Reduced depressive-like behaviors, reduced serum CORT levels, upregulation of GR and BDNF in the hippocampus	Zhang et al. (2022)
Rat pups exposed to DEX (6 h, single administration)	-	Upregulation of BDNF, NT-3 and NGF mRNA expression in the hippocampus.	Lanshakov et al. (2021)
Animals exposed to the CMS procedure for a period of 2 consecutive weeks.	Comparing anhedonic-like behavior (CMS vulnerable) and non anhedonia (CMS resilient)	Resilience to 2 weeks of CMS is paralleled by the activation of GRβ-RACK1 (scaffolding protein)-BDNF signaling in the ventral hippocampus	Brivio et al. (2021)
Presurgical exposure of mice to CUS	-	upregulation of GR, and downregulation of PKA and BDNF, and decreased ratio of pCREB/CREB levels in anterior cingulate and insular cortex, amygdala, dorsal horn, and dorsal root ganglion	Sun et al. (2023)
Spinal cord injury caused by severe mid-thoracic contusion of the spinal cord	-	Increased serum levels of corticosterone and decreased protein expression of hippocampal GR, BDNF, TrkB, and DCX	Liu et al. (2021)

Recent study demonstrates that an inhibition of FK506-binding protein 51 (FKBP51, a co-chaperone of GR) results in enhancement of neurite outgrowth of neurons and prevents depressive behaviors in animal model (Codagnone et al., 2022). Codagnone et al. (2022) found that SAFit2, a novel selective inhibitor of FKBP51, increased hippocampal neurite outgrowth and the number of branching nodes to a greater extent than BDNF in cultured hippocampal neurons. *In vivo* analysis also revealed that chronic administration of SAFit2 prevented stress-induced social avoidance and anxiety (but not alter adult hippocampal neurogenesis) in stressed animals (Codagnone et al., 2022). Interestingly, SAFit2 does not affect an increase in plasma corticosterone concentration after the chronic stress paradigm (Codagnone et al., 2022). Zhang et al. (2022) has reported the effects of formononetin (FMN, one of isoflavones, traditional Chinese medicine) on the depressive behaviors in chronic CORT injection mice (Zhang et al., 2022). In their system, it was revealed that decreased sucrose preference and increased immobility in FST in the depression mice were improved by treatment with FMN. Furthermore, reduced serum CORT levels, upregulation of GR and BDNF in the hippocampal region were also induced by FMN (Zhang et al., 2022). Using P2 rat pups exposed to DEX, long-lasting neurodevelopmental influence of glucocorticoid stress has been reported (Lanshakov et al., 2021). Single administration of DEX (6 h) caused upregulation of BDNF, NT-3 and NGF mRNA expression in the hippocampus. Interestingly, it was revealed that the upregulation of neurotrophins in the hippocampus and the downregulation of receptors in the brainstem Locus Coereleus norepinephrine neurons (NE) were observed. Furthermore, the elevated glucocorticoid level in the neonatal period decreases depressive-like behaviors (Lanshakov et al., 2021). As expected, it has been demonstrated that both glucocorticoid-and BDNF-mediated intracellular signaling are involved in the system of the vulnerability and resilience to chronic stress. Recent study suggests a relationship among the activity of HPA-axis, isoform alfa and beta of GR in the fundamental brain processes for the vulnerability and resilience to chronic stress (Brivio et al., 2021). It was reported that the resilience to 2 weeks of CMS is paralleled by the activation of GRβ-RACK1 (scaffolding protein)-BDNF signaling in the ventral hippocampus (Brivio et al., 2021). As mentioned above, most studies demonstrate downregulation of the BDNF/TrkB system by glucocorticoid stress and upregulation of the system in antidepressant treatment (Figure 2).

It has been demonstrated that pain sensitivity and existing pain are enhanced by chronic stress (Sun et al., 2023). Sun et al. (2023) examined influence of CUS on the pain induced by incisional surgery and found prolonged pain recovery after surgery by CUS. In their model, the upregulation of GR, and downregulation of PKA and BDNF, and decreased ratio of pCREB/CREB levels in several brain regions (anterior cingulate and insular cortex, amygdala, dorsal horn, and dorsal root ganglion) were also shown (Sun et al., 2023). Furthermore, Liu et al. (2021) investigated depressive-like behaviors after spinal cord injury (SCI) caused by severe mid-thoracic contusion of the spinal cord, and found that changes in an immobility time (FST) and sucrose preference were strongly correlated with levels of serum corticosterone. In their SCI models, significant increased serum levels of corticosterone and decreased protein expression of hippocampal GR, BDNF, TrkB, and DCX (Doublecortin, a microtubule-associated protein of neuronal precursor cells)-positive cells after SCI were observed (Liu et al., 2021), suggesting that depressive-like behaviors after SCI is also regulated via the HPA-axis in which GR/BDNF interaction is involved.

## 7. BDNF/TrkB system in AD

### 7.1. Relationship between BDNF/TrkB system and AD

Multiple studies have reported dysregulation of the BDNF/TrkB system in AD (Numakawa and Odaka, 2021, 2022; Numakawa and Kajihara, 2023). Garzon et al. (2002) showed that BDNF mRNA levels were lower in parietal cortex of AD patients (Garzon et al., 2002). A postmortem study of AD brains also found reduced protein levels of BDNF in the hippocampus and cortex (Du et al., 2018). In addition, another study reported decreased TrkB expression in the frontal and temporal cortex of AD brains (Allen et al., 1999).

Although the mechanisms underlying dysregulation of the BDNF/TrkB system in AD are not completely understood, various animal models were employed to investigate the molecular and cellular roles of the BDNF/TrkB system in the pathogenesis of AD. Psotta et al. (2015) generated a novel mouse model by crossing an established AD mouse model (APP/PS1) with a mouse strain characterized by a chronic deficiency of BDNF (BDNF^+/−^). The triple transgenic mice (APP/PS1/BDNF^+/−^) displayed an earlier onset of learning deficits and experienced accelerated impairment in a two-way active avoidance task, in contrast to the APP/PS1 or BDNF^+/−^ mice (Psotta et al., 2015). Likewise, through the breeding of BDNF^+/−^ mice with APPdE9 mice harboring APPswe and PSEN1ΔE9 mutations, another study demonstrated that the reduction in BDNF expression due to haploinsufficiency hindered learning and memory processes (Rantamäki et al., 2013). Recently, Wang et al. (2019) showed that BDNF knockout stimulated δ-secretase via activating JAK2/STAT3-C/EBPβ axis, leading to both APP and Tau fragmentation by δ-secretase and neuronal loss (Wang et al., 2019). They also reported that inhibition of JAK2/STAT3/C/EBPβ pathway prevented BDNF-depletion-mediated AD-like pathologies in a mouse model (Wang et al., 2019). These findings suggest that downregulation of the BDNF/TrkB system profoundly contributes to the pathophysiology of AD.

Upregulation of the BDNF/TrkB system has been shown to enhance neurogenesis, promote neuronal survival, and improve cognitive function in animal models of AD (Numakawa and Odaka, 2021, 2022; Numakawa and Kajihara, 2023). Using model mice expressing two APP mutations (Indiana (V717F) and Swedish (K670M)) associated with early-onset familial AD, Nagahara et al. (2013) demonstrated that lentiviral BDNF gene transfer into the entorhinal cortices of the mice at age 2 months prevented neuronal loss and improved hippocampal-dependent contextual fear conditioning (Nagahara et al., 2013). Similarly, restoration of the BDNF level by the intralateral ventricle injection of adeno-associated virus carrying the BDNF gene (AAV-BDNF) in the brains of mouse model expressing P301L mutant tau attenuated behavioral deficits, prevented neuron loss, alleviated synaptic degeneration, and reduced neuronal abnormality (Jiao et al., 2016). Another study also found that amelioration of neuronal atrophy and age-related cognitive impairment were observed by the administration of BDNF in rat AD brains (Nagahara et al., 2009). Moreover, the upregulation of BDNF has been found to reduce the production of toxic Aβ by facilitating the α-secretase processing of APP in transgenic APP/PS1 mice (Nigam et al., 2017). These observations suggest that BDNF treatment has the potential to directly modulate the amyloidogenic pathway, and that targeting the BDNF/TrkB system can be a promising therapeutic strategy for the disease.

In addition to AD, emerging evidence suggests that dysregulation of the BDNF/TrkB system also plays a crucial role in the pathophysiology of Parkinson’s disease (PD), another stress-related brain disease. PD is a progressive neurodegenerative disorder characterized by the loss of dopaminergic neurons in the substantia nigra (SN) and the presence of intracellular alpha-synuclein aggregates (Collins et al., 2012). Studies have reported reduced BDNF expression in the SN and other affected brain regions in PD patients (Palasz et al., 2020). The downregulation of BDNF may lead to inadequate neurotrophic support, impairing the survival and maintenance of dopaminergic neurons which are crucial for motor control. Furthermore, in the animal models of PD, restoring BDNF levels seemed to enhance dopaminergic neurons survival, induce dopaminergic axon regrowth, and elevate the dopamine level in the brains (Kim et al., 2012; Nam et al., 2015). Therefore, understanding the mechanisms underlying the dysregulation of the BDNF/TrkB system provides valuable insights into the neurodegenerative processes and potential therapeutic strategies not only for AD but also for PD.

### 7.2. Therapeutic interventions targeting BDNF/TrkB system in AD

Several approaches have been proposed to enhance BDNF/TrkB signaling, including direct administration of BDNF or its mimetics, and modulation of downstream signaling pathways that regulate TrkB expression (Table 2). A number of preclinical studies have investigated the efficacy of BDNF administration in animal models of AD, with promising results (Tapia-Arancibia et al., 2008; Nagahara and Tuszynski, 2011). However, challenges with blood–brain barrier (BBB) penetration have limited the clinical translation of BDNF as a therapeutic agent (Kopec et al., 2020; Fujisawa et al., 2022). Because of its high molecular weight (14 kDa), intravenous BDNF was not able to cross the BBB nor exert neuroprotective effects in APP/PS1 mice (Kopec et al., 2020). Therefore, several strategies need to be explored to enhance the delivery of BDNF across the BBB. These approaches include techniques such as the “Trojan Horse” delivery method (Pardridge, 2017), BBB modulators (Kopec et al., 2020), magnetic nano carriers (Pilakka-Kanthikeel et al., 2013) and ultrasound with microbubbles (Wang et al., 2021). These methods have demonstrated varying degrees of success in facilitating the transport of therapeutic molecules into the brain. As an example of molecular Trojan horse, BDNF was conjugated to a monoclonal antibody (MAb) against transferrin receptor, an endogenous BBB receptor transporter (Zhang and Pardridge, 2006). The intravenous administration of the BDNF-MAb conjugate was shown to cross the BBB, resulting in an improvement in neuro-behavior in a rat stroke model (Zhang and Pardridge, 2006).

**Table 2:** Therapeutic interventions targeting BDNF/TrkB system in AD models.

Experimental AD models	Treatments	Neurotrophic effects	References
Transgenic mouse expressing the APP Indiana (V717F) and Swedish (K670M) mutations	Lentiviral BDNF gene delivery into the entorhinal cortices at age 2 months	Prevention of neuronal loss and improvement in hippocampal-dependent contextual fear conditioning	Nagahara et al. (2013)
Transgenic mouse expressing P301L mutant tau	Intraventricular injections of AAV-BDNF at 3 months of age	Attenuation of behavioral deficits, neuronal loss, synaptic degeneration, and neuronal abnormality	Jiao et al. (2016)
Aged (24-month-old) Fischer rat	Continuous 4-week infusions (Alzet minipumps) of 120 ng BDNF per day in artificial cerebrospinal fluid	Amelioration of neuronal atrophy and age-related cognitive impairment	Nagahara et al. (2009)
C57BL/6 J mouse injected with scopolamine (1 mg/kg)	Oral administration of 150 mg/kg of YG per day for 14 weeks	Amelioration of memory impairment in the Y-maze, novel object recognition, and passive avoidance tests	Pak et al. (2022)
3xTg mouse	Daily oral injection of 3 mg/kg for CF3CN (TrkB receptor agonist)	Downregulation of brain Aβ levels and improvement of cognitive functions	Liao et al. (2021)
Rat exposed to high intraocular pressure by microbead injections	Intraperitoneal administration of 5 mg/kg 7,8 DHF for 8 weeks	Reduced Aβ levels	Gupta et al. (2022)
5XFAD mouse	Oral administration of R13 (7.25, 21.8, and 43.6 mg/kg) for 3 months	Prevention of Aβ deposition, mitigation of the loss of hippocampal synapses, and memory deficits	Chen et al. (2018)
Wistar rat	Voluntary running on a treadmill for 40 min daily for 6 days	Enhanced learning and memory ability and upregulation of both BDNF and TrkB	Xu et al. (2021)
APP+PS1 mouse	Voluntary running on a running wheel for 3 weeks	Upregulation of BDNF and α-secretase processing of APP, reduced production of toxic Aβ peptides	Nigam et al. (2017)
3xTg-AD mouse	Training on a rodent motor-driven treadmill with a frequency of 5 days per week for 12 weeks	Lower levels of Aβ plaque burden and neuro-inflammation, alleviation of mitochondrial dysfunction	Kim et al. (2019)

On the other hand, several small molecules that penetrate the BBB and modulate BDNF/TrkB signaling pathway have been identified as potential therapeutic agents for AD. These include 7,8-dihydroxyflavone (7,8-DHF), a selective TrkB agonist (Wurzelmann et al., 2017), and Yuk-Gunja-Tang (YG), a Korean traditional medicine which can increase the endogenous expression of BDNF (Pak et al., 2022). Several preclinical studies have demonstrated the efficacy of these agents in animal models of AD (Liao et al., 2021; Gupta et al., 2022; Lee et al., 2023). Recently, a prodrug of 7,8-DHF called R13 has been formulated with the aim of enhancing oral bioavailability and optimizing pharmacokinetic profiles (Chen et al., 2018). Dose-dependent administration of R13 through oral route in mice models of AD resulted in the activation of TrkB signaling. This activation effectively prevented Aβ deposition, mitigated the loss of hippocampal synapses, and alleviated memory deficits (Chen et al., 2018). The findings from these studies indicate the potential therapeutic value of 7,8-DHF and its derivatives for the treatment of AD. However, in order to consider their future clinical application, it is important to assess the safety and tolerability of these compounds in human subjects.

Exercise and environmental enrichment have been shown to upregulate the BDNF/TrkB system in the brain and promote neuroplasticity (Xu et al., 2021). These non-pharmacological approaches may hold promise as adjunctive therapies for AD. Several studies have investigated the effects of exercise on cognitive function in patients with AD (De la Rosa et al., 2020; Meng et al., 2020). A meta-analysis of randomized controlled trials found that exercise interventions were associated with significant improvements in global cognitive function, attention, and executive function in patients with mild-to-moderate AD (Jia et al., 2019). Similarly, studies have demonstrated that exercise training can improve cognitive function and promote neuroplasticity in animal models of AD (Nigam et al., 2017; Kim et al., 2019).

In conclusion, the BDNF/TrkB system plays an important role in AD pathology. Enhancing BDNF/TrkB system through pharmacological or non-pharmacological means may hold promise for the development of novel therapeutics for the disease.

## 8. The interplay between glucocorticoids, stress, and AD

### 8.1. Glucocorticoids and AD

**Figure 4:**
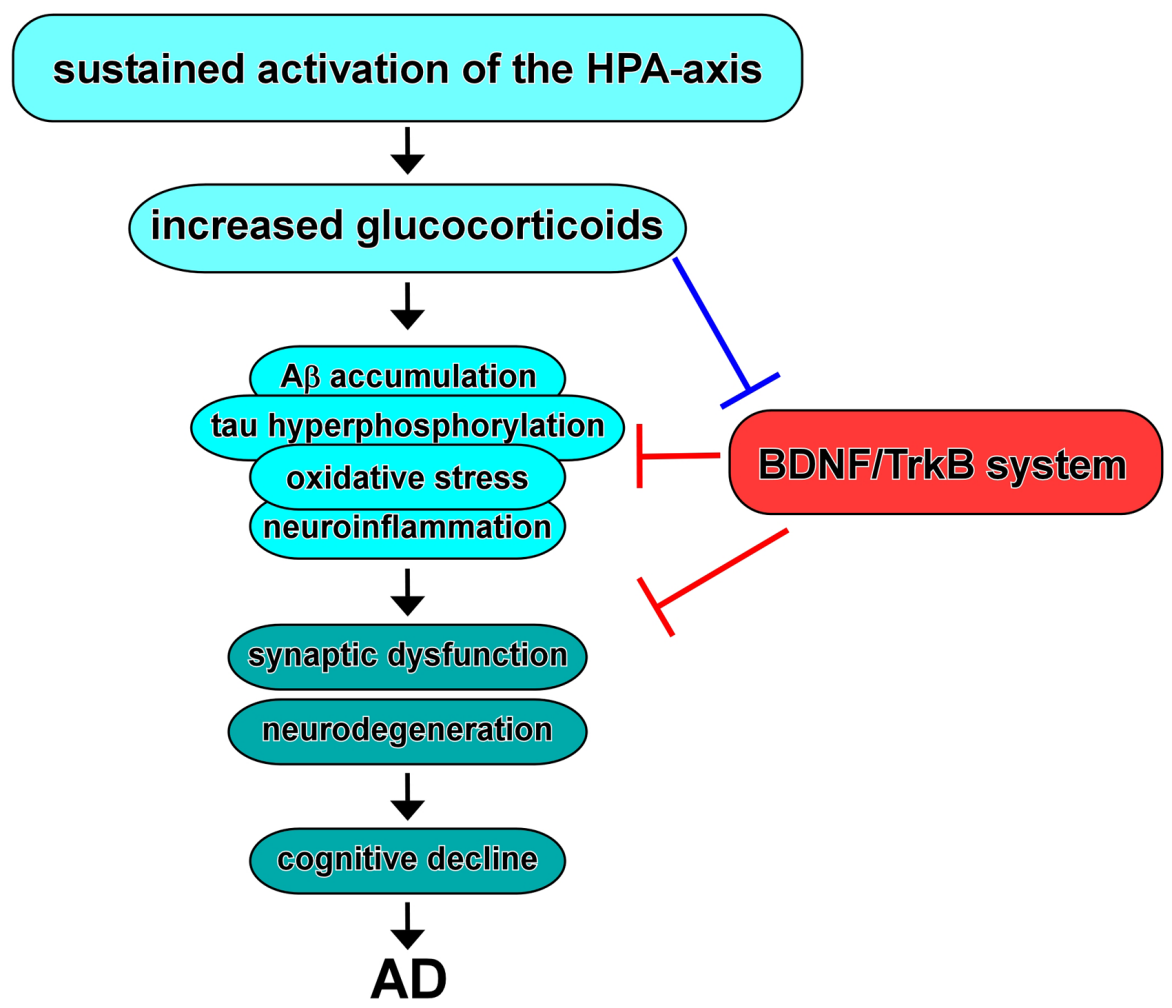
Dysregulation of the HPA-axis in AD and BDNF/TrkB system. Increased glucocorticoids due to sustained activation of the HPA-axis contribute to Aβ and/or tau pathology, oxidative stress, and neuroinflammation, resulting in synaptic dysfunction and neurodegeneration, which may link to cognitive decline observed in AD. Evidence suggest that increased glucocorticoids cause downregulation of expression/function of BDNF. In turn, upregulation of the BDNF/TrkB system improves the neurotoxicity in AD.

The role of glucocorticoids, the primary stress hormones, in AD has garnered significant attention in recent years (Figure 4). Glucocorticoids, primarily cortisol in humans, are released through activation of the HPA-axis in response to stress and play crucial roles in various physiological processes, including metabolism, immune regulation, and cognition (Beaupere et al., 2021; Shimba et al., 2021; Dekkers et al., 2022). In addition, dysregulation of the glucocorticoid system has been implicated in the pathogenesis and progression of AD (Caruso et al., 2019).

Studies have shown that glucocorticoids can influence key pathological features of AD, including Aβ accumulation and tau hyperphosphorylation (Zhuravleva et al., 2021). Glucocorticoids can enhance the generation of Aβ peptides by promoting the cleavage of APP and inhibiting Aβ clearance mechanisms (Wang et al., 2011; Yang et al., 2022). Additionally, glucocorticoids promote tau hyperphosphorylation, leading to the formation of neurofibrillary tangles (Monteiro-Fernandes et al., 2021). These effects suggest that glucocorticoids contribute to the initiation or exacerbation of AD pathology.

In addition to their impact on AD pathology, glucocorticoids play a role in neuroinflammation and synaptic plasticity, which are integral processes in AD pathogenesis. Glucocorticoids can modulate the immune response in the brain, influencing microglial activation, cytokine release, and oxidative stress (Pedrazzoli et al., 2019; Komoltsev and Gulyaeva, 2022; Correia et al., 2023). Chronic exposure to elevated glucocorticoid levels leads to a state of chronic neuroinflammation, promoting neuronal damage and impairing synaptic function (Hu et al., 2016; Zhang et al., 2017). Furthermore, glucocorticoids have been shown to affect synaptic plasticity, including long-term potentiation (LTP) and long-term depression (LTD), which are critical for learning and memory processes (Klyubin et al., 2022).

Epidemiological and clinical studies have provided evidence supporting the association between chronic exposure to glucocorticoids and an increased risk of AD development and cognitive decline. Long-term exposure of glucocorticoids, as seen in certain clinical conditions such as Cushing’s syndrome or prolonged use of corticosteroid medications, has been associated with cognitive impairment resembling aspects of AD (Forget et al., 2000; Notarianni, 2013). Moreover, individuals with a history of chronic stress or psychiatric disorders characterized by dysregulation of the HPA axis, which controls glucocorticoid release, may be more susceptible to AD pathology (Pomara et al., 2003; Gil-Bea et al., 2010).

Besides AD, several lines of evidence also suggest a complex relationship between glucocorticoids and PD pathology (van Wamelen et al., 2020). Previous studies also showed higher cortisol levels in PD patients (Soares et al., 2019). In rodent models, elevated levels of glucocorticoids have been shown to deteriorate motor performance and result in a more pronounced permanent loss of nigral neurons through mitochondrial malfunction and excessive oxidative stress (van Wamelen et al., 2020; Claros et al., 2021).

### 8.2. Dysregulation of the HPA-axis in AD

Growing evidence suggests that the HPA-axis is dysregulated in individuals with AD (Watermeyer et al., 2021). This dysregulation can manifest as alterations in baseline cortisol levels, blunted or exaggerated cortisol responses to stress, and disruption of the diurnal cortisol rhythm (Joshi and Praticò, 2013). It is hypothesized that dysregulation of the HPA-axis may contribute to the development and progression of AD by exposing the brain to chronic elevations of glucocorticoids (Canet et al., 2019; Milligan Armstrong et al., 2021).

Chronic stress, a prolonged state of heightened psychological and physiological arousal, has been implicated as a potential risk factor for AD. Chronic stressors, such as caregiving responsibilities, social isolation, or work-related stress, can lead to sustained activation of the HPA-axis, resulting in increased glucocorticoid release (Gupta, 2019). Elevated glucocorticoid levels, in turn, can exert detrimental effects on the brain, including impaired neuronal function, synaptic loss, and increased vulnerability to AD pathology (Pomara et al., 2003; Gil-Bea et al., 2010; Figure 4).

The relationship between stress and AD is bidirectional, with stress both influencing and being influenced by the pathophysiology of the disease. Chronic stress and glucocorticoid exposure can promote the accumulation of Aβ peptides by disrupting the balance between Aβ production, clearance, and aggregation (Wang et al., 2011; Yang et al., 2022). Conversely, Aβ accumulation can disrupt HPA-axis regulation, leading to altered stress responses and further exacerbation of neurodegeneration (Xu et al., 2018; Ruan et al., 2019). This bidirectional relationship highlights the intricate interplay between stress, glucocorticoids, and AD pathogenesis.

### 8.3. Mechanisms underlying glucocorticoid-induced neurotoxicity in AD

Glucocorticoid excess, especially under conditions of chronic stress, can lead to increased oxidative stress within the brain. Glucocorticoids have been shown to promote the production of reactive oxygen species (ROS) and impair the antioxidant defense system, resulting in oxidative damage to neurons (Claros et al., 2021). Furthermore, prolonged exposure to glucocorticoids can disrupt mitochondrial function, leading to energy deficits, impaired neuronal metabolism, and increased vulnerability to neurodegeneration (Choi and Han, 2021; Du et al., 2023).

Neuroinflammation is a prominent feature of AD pathology, and glucocorticoids have been implicated in modulating neuroinflammatory processes. Glucocorticoids can exert both anti-inflammatory and pro-inflammatory effects, depending on the context and duration of exposure (Bolshakov et al., 2021). Acute glucocorticoid administration may dampen neuroinflammation by suppressing pro-inflammatory cytokines (Ehrchen et al., 2019). However, chronic or excessive glucocorticoid exposure can promote a pro-inflammatory microenvironment, exacerbating neuronal damage and contributing to neurodegeneration (Hu et al., 2016; Zhang et al., 2017).

Moreover, glucocorticoids modulate synaptic plasticity in the hippocampus and other brain regions. While acute glucocorticoid exposure can enhance synaptic plasticity (Popoli et al., 2011), chronic or excessive glucocorticoid levels can impair LTP, disrupt synaptic connections, and contribute to cognitive deficits observed in AD (Klyubin et al., 2022). In addition, glucocorticoids have a role in the transcriptional regulation of BDNF gene via accessing the regulatory sequences present in the BDNF promoter region (Chen et al., 2017). Upon glucocorticoid binding to GRs, transcriptional repression of BDNF gene expression occurs (Chen et al., 2017). It has also been suggested that glucocorticoids reduce BDNF protein secretion, and affect the availability of BDNF in the extracellular environment and its interaction with TrkB receptors (Suri and Vaidya, 2013). Intracellular signaling pathways of the BDNF/TrkB system, such as the PLCγ, PI3K/Akt and MAPK cascades, are also known to be modulated by glucocorticoids, leading to altered activation of downstream effectors involved in structural and functional plasticity in AD-relevant brain regions and cognitive decline, one of the most common pathologies of AD (Numakawa et al., 2009, 2017).

For the direct impact of glucocorticoids on AD etiology, they have been shown to influence the production, clearance, and aggregation of Aβ peptides, which are key players in AD pathogenesis. Yang et al. (2022) reported that glucocorticoid enhanced the production of Aβ peptides and accelerated neuronal damage of hippocampal neurons in APP/PS1 mice (Yang et al., 2022). In addition, Wang et al. (2011) demonstrated that glucocorticoids markedly reduced Aβ degradation and clearance by mouse astrocytes due to the decreased expressions of several Aβ-degrading proteases, such as insulin-degrading enzyme and matrix metalloproteinase-9 (Wang et al., 2011). Moreover, another study demonstrated that glucocorticoids facilitated tau protein hyperphosphorylation, leading to the formation of neurofibrillary tangles, a hallmark of AD pathology (Monteiro-Fernandes et al., 2021).

The mechanisms underlying glucocorticoid-induced neurotoxicity in AD involve a complex interplay of oxidative stress, mitochondrial dysfunction, neuroinflammation, altered synaptic plasticity, and modulation of Aβ and tau pathology. These processes contribute to neuronal damage, synaptic loss, and cognitive decline observed in AD. Understanding the molecular pathways and signaling mechanisms involved in glucocorticoid-mediated neurotoxicity is crucial for identifying potential targets for therapeutic interventions aimed at mitigating the detrimental effects of glucocorticoids in AD. By modulating these mechanisms, it may be possible to attenuate neurodegeneration and improve cognitive outcomes in individuals affected by AD.

### 8.4. Therapeutic interventions targeting glucocorticoids in AD

Given the evidence linking glucocorticoids to AD pathology, targeting the dysregulation of the glucocorticoid system presents a potential avenue for therapeutic interventions (Table 3).

**Table 3:** Therapeutic interventions targeting glucocorticoids system in AD models.

Experimental AD models	Treatments	Neurotrophic effects	References
3xTg mouse	Subcutaneous administration of CORT108297 (1.2 mg per day) for 21 days	Suppression of the levels of APP C-terminal fragments	Baglietto-Vargas et al. (2013)
Sprague–Dawley rat injected intracerebroventricularly with Aβ25–35 peptide	Intraperitoneal injection of CORT108297 (20 mg/kg) and CORT113176 (10 mg/kg) for 1 week	Reversal of hippocampal amyloid-β peptide generation, neuroinflammation, and apoptotic processes, restoration of the hippocampal levels of synaptic markers, and cognitive function	Pineau et al. (2016)
Aged (24-month-old) C57BL/6 J mouse	Intraperitoneal administration of UE1961 (10 mg/kg, i.p.) twice daily for 10 days	Improvement of spatial memory performance in the Y-maze	Sooy et al. (2010)
Tg2576 mouse	Continuous infusions (Alzet minipumps) of UE2316 (10 mg/kg/day) for 29 days	Reduction of Aβ plaques in the cerebral cortex, upregulation of insulin-degrading enzyme (IDE) levels and memory improvements	Sooy et al. (2015)
Aged CD1/ICR mouse or Sprague–Dawley rat	Oral administration of A-918446 (1–100 mg/kg) and A-801195 (1–30 mg/kg) for 1 h	Improvement of memory consolidation and recall in inhibitory avoidance, and increment of CREB phosphorylation	Mohler et al. (2011)

Pharmacological strategies targeting glucocorticoids include the development of selective glucocorticoid receptor modulators (GRMs) that selectively block GR activity (Canet et al., 2019; Watermeyer et al., 2021). These compounds aim to maintain the beneficial anti-inflammatory effects of glucocorticoids while minimizing their detrimental impact on neuronal function. CORT108297, developed by Corcept Therapeutics (San Mateo, CA, United States), stands as one of the compounds. A study showed that treatment with CORT108297 suppressed the levels of APP C-terminal fragments in the 3xTg-AD mouse model (Baglietto-Vargas et al., 2013). Moreover, Pineau et al. (2016) investigated the therapeutic potential of CORT108297 and CORT113176 in an acute rat model of AD (Pineau et al., 2016). CORT113176 is another GRM with a higher affinity for GR compared to CORT108297. In the study, the mice administrated with CORT108297 and CORT113176 via intraperitoneal injection had a complete reversal of memory deficits as assessed by the T maze test (Pineau et al., 2016).

In addition to the GRMs, compounds that interfere with glucocorticoid synthesis or promote glucocorticoid clearance may be explored as potential therapeutic options (Canet et al., 2019; Watermeyer et al., 2021). 11β-HSD1 plays a critical role as an enzyme responsible for the intracellular conversion of inactive cortisone into active cortisol in human (11-dehydrocortisone to corticosterone in rodents). Therefore, the inhibition of 11β-HSD1 leads to a reduction in cortisol levels in humans (or corticosterone levels in rodents; Canet et al., 2019; Watermeyer et al., 2021). Sooy et al. (2010) showed that the treatment of UE1961, a 11β-HSD1 inhibitor, significantly improved spatial memory performance in aged mice (Sooy et al., 2010). Furthermore, they also demonstrated that the use of another inhibitor, UE2316, led to a reduction in Aβ plaques in the cortex of aged Tg2576 mice. This reduction was accompanied by an increase in insulin-degrading enzyme levels and resulted in memory improvements (Sooy et al., 2015). Another study also characterized two novel and selective 11β-HSD1 inhibitors, A-918446 and A-801195. The inhibitors improved memory consolidation and recall in inhibitory avoidance and increased CREB phosphorylation in the cingulate cortex of rodent models (Mohler et al., 2011).

Non-pharmacological interventions that promote resilience to stress and enhance cognitive function may be beneficial in AD management. Cognitive training programs, including memory exercises and cognitive stimulation, have shown promising results in improving cognitive abilities and quality of life in individuals with AD (Irazoki et al., 2020). Moreover, interventions that foster social support, engagement in meaningful activities, and emotional well-being may help counteract the detrimental effects of chronic stress and glucocorticoid dysregulation. Lifestyle modifications that reduce chronic stress levels also have a positive impact on AD progression. Stress management techniques such as mindfulness-based stress reduction, cognitive-behavioral therapy, and regular physical exercise have been shown to reduce cortisol levels and improve cognitive function in individuals at risk for AD (Watermeyer et al., 2021). Promoting a healthy lifestyle that includes adequate sleep, a balanced diet, and social engagement can contribute to stress reduction and overall brain health.

While therapeutic interventions specifically targeting glucocorticoids in AD are still in their early stages, understanding the potential of modulating the glucocorticoid system provides a novel avenue for intervention. Further preclinical and clinical research is needed to evaluate the safety, efficacy, and long-term benefits of these approaches. By targeting glucocorticoid dysregulation and mitigating its neurotoxic effects, it may be possible to develop more effective treatments that slow down or prevent the progression of AD, ultimately improving the quality of life for individuals affected by this devastating disease.

## 9. Conclusion

Stress-related brain diseases, including MDD and AD, have become a significant global health concern due to their devastating impact on individuals and societies. MDD is characterized by persistent feelings of sadness, hopelessness, loss of interest or pleasure in activities, and disturbances in cognitive functions. AD, characterized by progressive cognitive decline and memory impairment, is the most common form of dementia. It is estimated that the number of individuals affected by the disease will continue to rise as the population ages. The exact etiologies of both MDD and AD remain elusive, but they are widely recognized to be multifactorial, involving a complex interplay between genetic, environmental, and neurobiological factors.

Recent research has shed light on the intricate interplay between BDNF-signaling and stress in the pathogenesis of MDD and AD. BDNF, essential growth factor for neuronal survival, development, synaptic plasticity in CNS neurons, is involved in memory and emotional processing. Stress, both acute and chronic, has long been recognized as a significant risk factor for the development of both MDD and AD. Stressful life events can trigger depressive episodes, and individuals with MDD or AD often exhibit dysregulated stress responses. The intricate relationship between stress, MDD and AD is thought to involve alterations in neurobiological pathways, including those influenced by the BDNF/TrkB system.

Glucocorticoids, as stress hormones, play a critical role in the body’s stress response and have been implicated in the pathophysiology of AD. Chronic or excessive glucocorticoid exposure is associated with adverse effects on various physiological systems, including the brain function. Glucocorticoids can modulate inflammatory responses, oxidative stress, and deficits in synaptic plasticity, all of which are implicated in the progression of AD. Furthermore, increased glucocorticoids directly exacerbate the accumulation of Aβ plaques and tau tangles, the hallmark pathological features of AD. The negative impact of glucocorticoid stress on cognitive function and AD risk is an area of active research and continues to yield valuable insights.

Considering the therapeutic implications, targeting BDNF signaling pathways holds promise as a potential strategy for the management of stress-related brain diseases. Pharmacological interventions aimed at restoring BDNF levels or enhancing BDNF signaling have shown potential in preclinical and clinical studies. Non-pharmacological approaches, such as cognitive-behavioral therapies and physical exercise, have also demonstrated positive effects on BDNF levels and may serve as adjunctive treatments for the diseases although several challenges and research gaps still remain. For example, safety concerns and potential side effects associated with BDNF intervention must also be carefully considered. As BDNF has pleiotropic effects on various cell types in the CNS with peripheral tissues through activating a variety of signaling pathways, achieving the desired therapeutic effects without triggering adverse reactions can be challenging. As shown above, activation of the BDNF/TrkB system is estimated to be affected by contribution of various types of TrkB receptors (full length, truncated, and so on), interactors including p75NTR, and conversion of proBDNF (primally for p75NTR) to mature BDNF (for full length TrkB), therefore, specific reinforcing the BDNF/TrkB system to achieve an increased neuronal function should be carefully examined. Recently, available evidence indicates BDNF has a role in the regulation of energy balance as both BDNF and receptors are extensively expressed in the hypothalamus that controls feeding and metabolism activation, and is involved in anorexigenic and orexigenic effects (Di Rosa et al., 2021), suggesting that regulation of the BDNF/TrkB system in the CNS may also influence on the peripheral tissues including liver and the adipose tissue, resulting in affecting obesity-related diseases. The variability in BDNF response among individuals with the disorders is another limitation. Factors such as genetic variations, disease stage, and other comorbidities can influence the efficacy of BDNF treatment. Therefore, further investigations are needed to unravel the precise molecular mechanisms underlying BDNF dysregulation in MDD and AD, as well as the intricate interactions between BDNF, stress, and other neurobiological pathways implicated in these disorders. Longitudinal studies examining the effects of interventions targeting BDNF signaling in the patients are warranted to establish their efficacy and safety.

Overall, this comprehensive review contributes to the growing body of knowledge in the field of stress-related brain diseases, providing a foundation for further research, clinical practice, and the development of innovative interventions that capitalize on the potential of BDNF/TrkB system modulation in the management of these challenging disorders.

## Author contributions

TN and RK wrote and edited the paper. All authors contributed to the article and approved the submitted version.

## Funding

This study was supported by grants from the Grant-in-Aid for Scientific Research (C) (JSPS KAKENHI 20K06857) (TN) of the Ministry of Education, Culture, Sports, Science, and Technology of Japan, and from the Takeda Science Foundation (TN). This study was also supported by Japanese Society of Inherited Metabolic Disease/Sanofi LSD Research Grant (RK), and the Grant-in-Aid for Young Scientists (JSPS KAKENHI 23K07772) (RK).

## Conflict of interest

The authors declare that the research was conducted in the absence of any commercial or financial relationships that could be construed as a potential conflict of interest.

## Publisher’s note

All claims expressed in this article are solely those of the authors and do not necessarily represent those of their affiliated organizations, or those of the publisher, the editors and the reviewers. Any product that may be evaluated in this article, or claim that may be made by its manufacturer, is not guaranteed or endorsed by the publisher.
